# Supplementary material for: Causal Relationship between Aging and Anorexia Nervosa: A White-Matter-Microstructure-Mediated Mendelian Randomization Analysis
Source: Biomedicines. 2024 Aug 16;12(8):1874. doi: 10.3390/biomedicines12081874 (PMC11351342; doi:10.3390/biomedicines12081874)
Supplement: Supplementary file 1 [file biomedicines-12-01874-s001.zip › Supplementary Figure/Figure S4/Singe SNP plot of MR analysis for TL to ubm-b-2109.pdf]

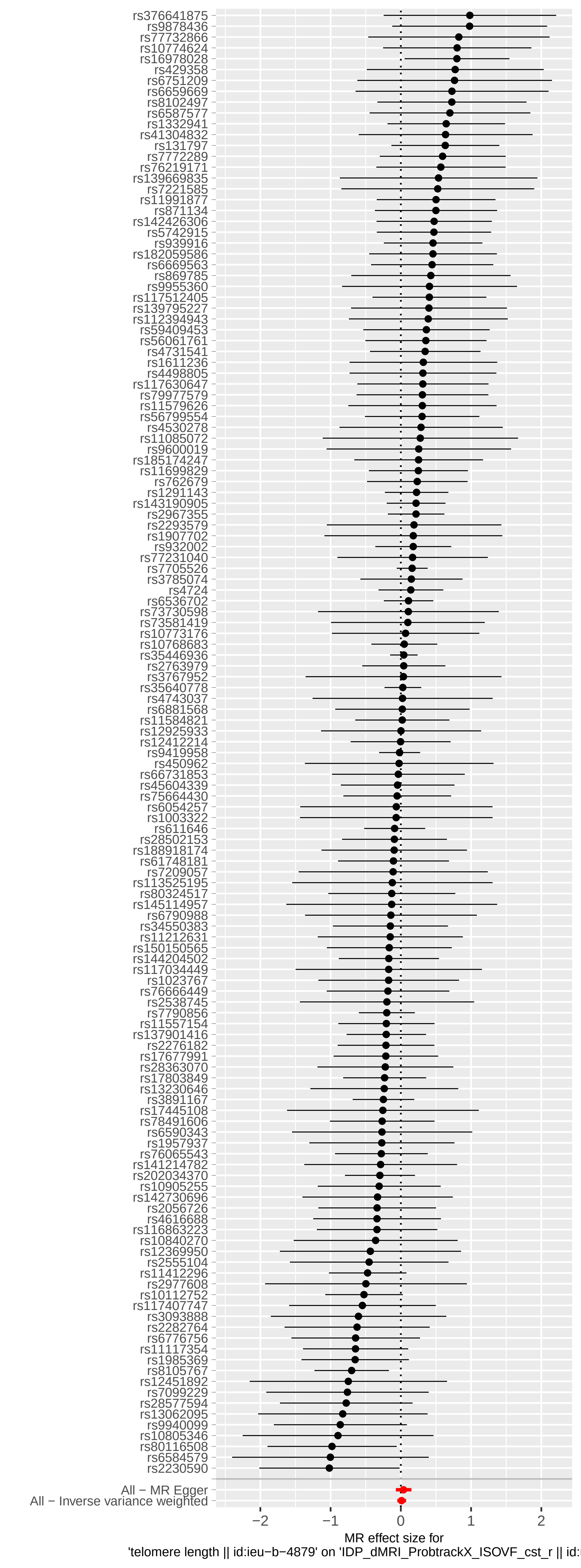
rs376641875
rs9878436
rs77732866
rs10774624
rs16978028
rs429358
rs6751209
rs6659669
rs8102497
rs6587577
rs1332941
rs41304832
rs131797
rs7772289
rs76219171
rs139669835
rs7221585
rs11991877
rs871134
rs142426306
rs5742915
rs939916
rs182059586
rs6669563
rs869785
rs9955360
rs117512405
rs139795227
rs112394943
rs59409453
rs56061761
rs4731541
rs1611236
rs4498805
rs117630647
rs79977579
rs11579626
rs56799554
rs4530278
rs11085072
rs9600019
rs185174247
rs11699829
rs762679
rs1291143
rs143190905
rs2967355
rs2293579
rs1907702
rs932002
rs77231040
rs7705526
rs3785074
rs4724
rs6536702
rs73730598
rs73581419
rs10773176
rs10768683
rs35446936
rs2763979
rs3767952
rs35640778
rs4743037
rs6881568
rs11584821
rs12925933
rs12412214
rs9419958
rs450962
rs66731853
rs45604339
rs75664430
rs6054257
rs1003322
rs611646
rs28502153
rs188918174
rs61748181
rs7209057
rs113525195
rs80324517
rs145114957
rs6790988
rs34550383
rs11212631
rs150150565
rs144204502
rs117034449
rs1023767
rs76666449
rs2538745
rs7790856
rs11557154
rs137901416
rs2276182
rs17677991
rs28363070
rs17803849
rs13230646
rs3891167
rs17445108
rs78491606
rs6590343
rs1957937
rs76065543
rs141214782
rs202034370
rs10905255
rs142730696
rs2056726
rs4616688
rs116863223
rs10840270
rs12369950
rs2555104
rs11412296
rs2977608
rs10112752
rs117407747
rs3093888
rs2282764
rs6776756
rs11117354
rs1985369
rs8105767
rs12451892
rs7099229
rs28577594
rs13062095
rs9940099
rs10805346
rs80116508
rs6584579
rs2230590
All − MR Egger
All − Inverse variance weighted
−2
−1
0
1
2
MR effect size for
'telomere length || id:ieu−b−4879' on 'IDP_dMRI_ProbtrackX_ISOVF_cst_r || id:
